# Salivary glands adenoid cystic carcinoma: a molecular profile update and potential implications

**DOI:** 10.3389/fonc.2023.1191218

**Published:** 2023-07-05

**Authors:** Fernanda Jardim da Silva, Juscelino Carvalho de Azevedo, Ana Carolina Lima Ralph, João de Jesus Viana Pinheiro, Vanessa Morais Freitas, Danielle Queiroz Calcagno

**Affiliations:** ^1^ Núcleo de Pesquisas em Oncologia, Programa de Pós-Graduação em Oncologia e Ciências Médicas, Universidade Federal do Pará, Belém, Brazil; ^2^ Hospital Universitário João de Barros Barreto, Programa de Residência Multiprofissional em Saúde (Oncologia), Universidade Federal do Pará, Belém, Brazil; ^3^ Faculdade de Farmácia, Faculdade Estácio, Carapicuíba, Brazil; ^4^ Instituto de Ciências da Saúde, Programa de Pós-Graduação em Odontologia, Universidade Federal do Pará, Belém, Brazil; ^5^ Laboratório de Microambiente Tumoral, Instituto de Ciências Biomédicas, Universidade de São Paulo, São Paulo, Brazil

**Keywords:** adenoid cystic carcinoma, oral cancer, molecular profile, target therapies, MYB

## Abstract

Adenoid cystic carcinoma (ACC) is an aggressive tumor with a high propensity for distant metastasis and perineural invasion. This tumor is more commonly found in regions of the head and neck, mainly the salivary glands. In general, the primary treatment modality for ACC is surgical resection and, in some cases, postoperative radiotherapy. However, no effective systemic treatment is available for patients with advanced disease. Furthermore, this tumor type is characterized by recurrent molecular alterations, especially rearrangements involving the *MYB, MYBL1*, and *NFIB* genes. In addition, they also reported copy number alterations (CNAs) that impact genes. One of them is *C-KIT*, mutations that affect signaling pathways such as NOTCH, PI3KCA, and PTEN, as well as alterations in chromatin remodeling genes. The identification of new molecular targets enables the development of specific therapies. Despite ongoing investigations into immunotherapy, tyrosine kinase inhibitors, and anti-angiogenics, no systemic therapy is approved by the FDA for ACC. In this review, we report the genetic and cytogenetic findings on head and neck ACC, highlighting possible targets for therapeutic interventions.

## Background

Adenoid cystic carcinoma (ACC) is a rare and aggressive tumor mainly affecting salivary glands (1). Cytologically, the ACC comprises myoepithelial and luminal cells and is histologically classified into tubular, cribriform, or solid subtypes (2). The solid subtype is the most aggressive and is prone to poor prognosis and a higher frequency of mutations (3, 4).

According to histological patterns, ACC can be classified into high and low grades. Low-grade ACC consists of grades I (predominantly tubular, no solid areas or occasionally solid areas) and II (predominantly cribriform, <30% solid). High-grade ACC is defined as grade III (>30% solid component) (5, 6). It is common for tumors to show transitions between the three histological patterns (5).

The grade III solid histological pattern can be confused with high-grade transformation adenoid cystic carcinoma (ACC-HGT) due to some similarities with cellular atypia, occasional comedoiform necrosis, and frequent mitotic figures. However, the cells show a different aspect (solid-type cells: basaloid, small, hyperchromatic nuclei with scarce cytoplasm. ACC-HGT: larger, more pleomorphic, and vesicular nuclei and balance between nucleus and cytoplasm) (7). Some other aspects differentiate ACC from the solid subtype of ACC-HGT, which is highly prone to lymph node metastases, high rates of mitotic labeling and increased expression of Ki-67, as well as high expression of p53 (7, 8).

ACC is more frequently found in women in their fifth or sixth decade of life (1). Although ACC is usually slow growing, it is a very aggressive tumor with a progressive infiltrative growth pattern and a high propensity for perineural invasion, local recurrence, and distant metastasis, especially in the lung, bones, and liver (9–13). Depending on the clinical stage, different therapeutic approaches can be used for ACC, but surgical intervention with radiotherapy remains the main treatment strategy in the management of ACC. In addition, chemotherapy, targeted therapy, and immunotherapy have also been used to improve the survival and quality of life of patients with ACC (14). Unfortunately, no specific therapies are approved by the food and drug administration (FDA) to treat patients with recurrent or metastatic ACC (15). Knowing the molecular alterations of the tumor, as well as the interaction with the tumor microenvironment, is essential in the identification and development of new therapies (16).

Previous reports have demonstrated that molecular alterations in ACC are characterized mainly by fusions involving the MYB protein family (17). Furthermore, other molecular changes involve copy number alterations (CNAs) and mutations that have a relevant role in the progression of this disease, including *RAS*, *PTEN*, *PIK3CA*, and *NOTCH1-4* (3, 16). It is still unclear whether these alterations have any clinical impact.

In the personalized medicine era, it is essential to identify molecular alterations that can play an auxiliary role in therapeutic decision-making, thereby proving their utility as biomarkers for therapy response and clinical management in ACC patients (18). In this review, we focused on reporting the most relevant and recurrent molecular alterations described in ACC of salivary glands and their implications. Moreover, we discussed the clinical trials conducted to evaluate targeted therapies for ACC.

## Molecular alterations in ACC

### MYB/MYBL1 alterations

In ACC, the most commonly reported molecular alteration is the t(6;9) translocation, which results in the fusion of the 5′-end of *MYB* to the 3′-end of *NFIB* (17, 19–21). MYB protein belongs to a family of transcription factors composed of two other members: *MYBL1* and *MYBL2*. This family consists of three functional domains: the C-terminal negative regulatory domain (NRD); the N-terminal DNA binding domain, which is essential for interaction with the *MYB* binding site; and a centrally located transcription activation domain. The transactivation of *MYB* is also influenced by interaction with CBP/p300 and p100 coactivators (22). *NFIB* is a transcription factor involved in various physiological processes, including adipocyte differentiation, maturation of megakaryocytes, and brain and lung development (23).

**Figure 1:**
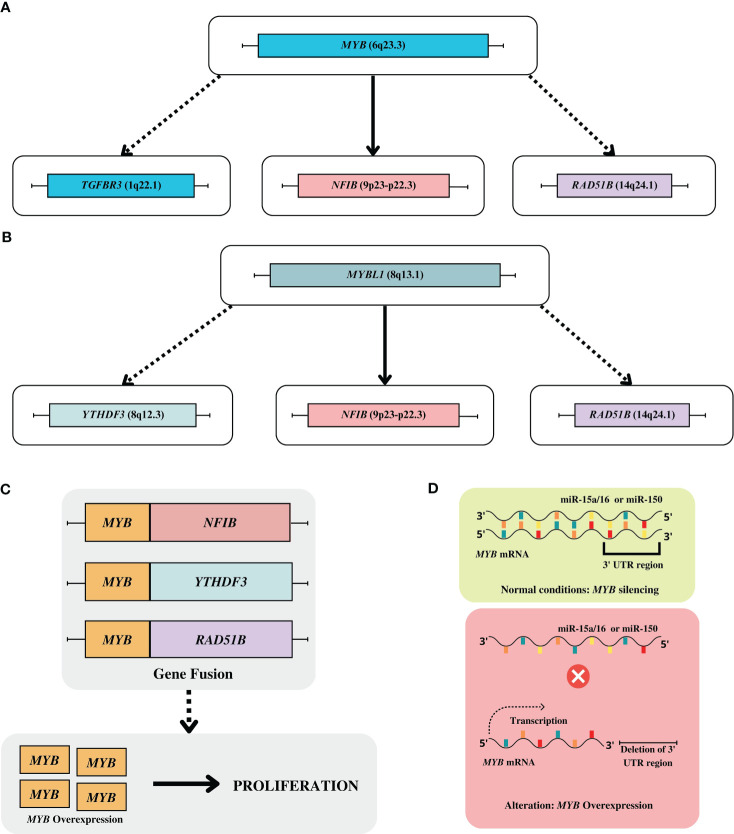
**(A)** Main gene fusions of *MYB* gene. **(B)** Main gene fusions of *MYBL1* gene. **(C)** Gene fusion of *MYB* with the super enhancers including *YTHDF3*, *RAD51B*; **(D)** Alteration on regulation of MYB expression: in normal conditions the *MYB* is downregulated by miR-15a/16 or miR-150, however in ACC tumor occur the deletion of 3’ UTR region blocking the ligation of their miRNA, which facilities the overexpression of *MYB* gene.

The frequency of *MYB-NFIB* fusion is variable (16% to 100%), depending on the approach used for analysis (24). Variability in *MYB-NFIB* fusion transcripts is frequently observed, mainly due to different breakpoints in both genes (19–21). Generally, most variants involve the fusion of *MYB* exon 14 to *NFIB* exon 9 (**Figure 1A**) (19–21). This diversity can result in full-length and truncated MYB proteins and fusion transcripts. Most truncations occur in the C-terminal domain, and there may also be retained DNA-binding and transactivation domains, suggesting that MYB may act to regulate gene expression in ACC (25).

These fusions usually result in the overexpression of MYB protein, and its target genes associated with various cellular activities, such apoptosis (*API5, BCL2, BIRC3, HSPA8, SET*), cell cycle control (*CCND1, CCNB1, CDC2, MAD1L1, MET*), cell growth/angiogenesis (*MYC, KIT, VEGFA, FGF2, CD53, FGFR2, IGF1R*), and cell adhesion (*CD34*) (19, 26–28). Notably, MYC overexpression associated with MYB overexpression is associated with a more advanced clinical stage and shorter disease-free survival (DFS) (29).

Possible downstream effects of MYB overexpression were also investigated. In addition to the target genes already described, other factors or transcription pathways may act on the ACC in a coordinated/mediated means by MYB. One such factor is TP63, which can co-bind with MYB at about 81% of binding sites. Furthermore, at binding sites close to MYB, the *EN1, ARID1A*, and *NOTCH1* genes, and their JAG1 and JAG2 activators are also identified as targets. MYB coordination programs with NOTCH and TP63 also play a role in the transition from grade 2 to grade 3 tumors. While TP63 is strongly expressed in myoepithelial cells from grade 2 tumors, luminal cells showed a high expression of ICN1 (form an active intracellular layer of NOTCH1) in a mutually exclusive manner. In contrast, grade 3 ACCs show strong staining for ICN1 (28). This corroborates the fact that MYB protein is highly expressed in myoepithelial cells (30).

The cell proliferation through *MYB-NFIB* in the ACC has complex activation mechanisms. Recently, it was demonstrated that cellular stress induced by MYB overexpression leads to a DNA damage response *via* the ATR pathway (31). Furthermore, *MYB-NFIB* is regulated by AKT-dependent IGF1R signaling (27).

In addition to the previously reported fusions, other mechanisms that lead to overexpression of MYB have been described. Through whole-genome sequencing (WGS) technology, it was possible to identify alternative mechanisms by which *MYB-TGFBR3* and *MYB-RAD51B* rearrangements can induce MYB overexpression. Such translocations result in the juxtaposition of super enhancers to the MYB locus, increasing their expression levels. In this context, enhancers at the *NFIB* and *TGFBR3* loci are notable since they can interact with the MYB protein, resulting in a positive feedback mechanism that promotes overexpression of MYB (**Figure 1C**) (28). Subsequently, a fluorescent *in situ* hybridization (FISH) analysis with wide-ranging probes revealed new breakpoints at the *MYB* locus that were distant from the *MYB* gene (~10 Mb), reinforcing the role of distant superenhancing elements in the overexpression of the protein (25). Finally, the deletion of the 3′ untranslated regions (3’UTR) of *MYB* prevents the silencing of this gene by miRNAs (miR-15a/16 and miR-150) (**Figure 1D**) (19).

Additionally, another gene from this family of transcription factors also plays a critical role in the development of ACC. It has previously been observed that the MYB protein can be expressed in the ACC regardless of the status of the *MYB-NFIB* fusion. This led to the consideration that there might be other MYB activation mechanisms (30). Two simultaneous and independently performed studies confirmed this hypothesis and found the presence of translocations involving *MYBL1*. Novel translocations in the 8q13 and 9p23 regions were identified, resulting in the *MYBL1-NFIB* fusion and rearrangements resulting in the *MYBL1–YTHDF3* fusion. Both cytogenetic alterations can lead to overexpression of MYBL1. An interesting observation is that analysis RT-PCR also detected high levels of MYBL1 mRNA in t(6;9) and t(8;9) negative tumors. Further analysis revealed the presence of truncated MYBL1 proteins due to rearrangements of adjacent intragenic sites on chromosome 8 and a chromosomal translocation between *MYBL1* and *RAD51B* (**Figure 1B**). The alterations in MYB and MYBL1 appear mutually exclusive (26, 32). Other reports also observed changes in *MYBL1*, reinforcing the driver gene’s role in ACC (33, 34).

### Clinical applicability

The clinical significance of fusions in members of the MYB family still needs to be well established (35–37). However, the *MYB-NFIB* fusion is specific for ACC (36, 37). Therefore, determining the status of the *MYB-NFIB* fusion could be useful for the differential diagnosis of ACC and other head and neck neoplasms for the subpopulation of patients with the fusion (38–40). In turn, overexpression of the MYB protein may be useful as a diagnostic biomarker due to its high specificity and sensitivity for ACC (36, 37, 41). It should be noted that the specific cutoff points for MYB immunoreactivity by immunohistochemistry (ICH) and the antibody used, which would be more useful to distinguish ACC, must be carefully defined (30, 36, 37, 41).

Likewise, the diversity of transcripts and multiple breakpoints require that the tool used for identification be carefully chosen (19–21). Issues such as primer design (PCR), probe coverage (FISH) and type of material [fixed in formalin and embedded in paraffin (FFPE) versus frozen] may influence the detection of changes (24). Investigation of *MYB-NFIB* transcription by RT-PCR showed significant differences in the percentage of positive cases in relation to frozen tissue (86%) and FFPE (44%) (30). Furthermore, most studies rely on RT-PCR to identify fusion transcripts or FISH analysis for the translocation. In this sense, both the primer designs and the cut-off value to determine the positivity of the FISH translocation is variable, which may impact the ability to detect the fusion (19–22). Notably, the high cost and complexity are some of the barriers that make it difficult to include more comprehensive and specific methods in the clinical routine, such as Next Generation Sequencing (NGS) and RNA *in situ* hybridization (ISH), evaluating the *MYB-NFIB* fusion routine a standard in the ACC cases a challenge (39, 40).

### Critical copy number alterations (CNAs)

In general, the patterns of chromosomal alterations in ACC are heterogeneous due to the multiple technologies used, differences in resolution, tested loci, and rigor of the criteria used to define gains or losses (24, 42).

Notably, CNAs have also been reported in ACC. Among the CNAs, the 6q region gain is the most frequently observed event (3, 43, 44). Furthermore, deletion and loss of heterozygosity (LOH) in this region were associated with locally advanced disease and cribriform histologic subtype, indicating that this event may be related to tumor progression (44, 45). The main candidate tumor suppressor genes in this region are *PLAGL1* and *LAST1*. However, was not observed inactivating mutations or evidence of gene silencing at the RNA and protein levels of these genes in ACC (46). On the other hand, in a recent analysis, it was noticed that the deletion of 6q distal to the *MYB* breakpoint at 6q23.3 is a concomitant event with the loss of the 3’ *MYB* region, which results in the subsequent t(6;9) translocation (17)

Gains in the 4q12 region and gene amplifications have been reported, events that may lead to overexpression of *C-KIT* (47–53). *C-KIT* is a gene that encodes a Type III tyrosine kinase receptor and plays an essential role in several biological functions, such as survival, metabolism, proliferation, apoptosis, and differentiation (54).

The expression pattern of *C-KIT* differs according to histological subtype, although some results are controversial. Most reports indicate strong *C-KIT* staining in the solid histological subtype (49–51). On the other hand, a median staining intensity of the solid phenotype about the cribriform and tubular subtypes was reported. However, cribriform and tubular subtypes may evolve into a solid phenotype during disease progression, suggesting that overexpression of C-KIT may be a key event for tumor progression (52).

This finding is consistent with a higher expression of *C-KIT* in tumor stages III-IV in patients with perineural invasion, regional local recurrence, and distant metastasis and is related to a poor prognosis (50, 55). Interestingly, *C-KIT* expression is positively correlated with stem cell factor (SCF) in ACC tumor cells and other tumor microenvironment cells, particularly in nerve cells, which may be associated with the strong trend of perineural invasion for this tumor (55).

Finally, ACC tumors also have a considerable number of cytogenetic deletions affecting genes coding primarily suppressor tumor genes and adhesion molecules, which regulate the cell cycle, resulting in excessive proliferation and contributing to the metastatic process (43, 47, 56). The main findings about the ACC genetic profile are summarized in **Table 1**.

**Table 1:** Recurrent CNAs in ACC.

Reference	Method	Samples	Alteration	Genes
Bernheim et al., 2008 (47)	aCGH, FISH	Frozen samples	Gain	*HOXA, MDM2, KIT*
			Loss	*CDKN2A/CDKN2B, TP53, LIMA1*
Rao et al, 2008 (56)	aCGH, FISH	Frozen samples	Loss	*P73, CDH5*
Persson et al., 2009 (19)	RT-PCR, FISH	Frozen samples, primary culture	Translocation	*MYB-NFIB*
Mitani et al., 2010 (20)	RT-PCR, FISH	Frozen samples	Translocation	*MYB-NFIB*
Mitani et al., 2011 (21)	RT-PCR, FISH	Frozen samples	Translocation	*MYB-NFIB*
Brill et al., 2011 (30)	RT-PCR	Frozen samples, FFPE	Transcripits Fusion	*MYB-NFIB*
Oga et al., 2011 (43)	aCGH	Frozen samples	Amplification	*MDM2, CPM, SLC35E3RAP1B, NUP107*
			Gain	*SYN3, TIMP3*
			Loss	*MDM2, CPM, SLC35E3RAP1B, NUP107*
West et al., 2011 (35)	FISH	Frozen samples	Translocation	*MYB-NFIB*
Persson et al., 2012 (57)	aCGH	Frozen samples	Gain	*PARK2, LATS, CASP9, CTNNBIP1, UBE4B, RUNX3, SFN, NBL1, PRDM2*
			Loss	*NR4A1, LIMA1*
Costa et al., 2014 (58)	aCGH, FISH	FFPE	Gain	*NFIB*
			Deletion	*ALDH8A1, HBS1L*
			Translocation	*MYB-NFIB*
Li et al., 2014 (59)	G-band analysis, aCGH	Frozen samples, primary culture	Translocation	**
			Gain	**
			Focal deletion	**
Ho et al., 2013 (3)	FISH	Frozen samples	Translocation	*MYB-NFIB*
Argyris et al., 2016 (60)	FISH	FFPE	Rearrangement	*MYB*
Brayer et al., 2016 (26)	RNA-seq, RT-PCR	FFPE	Translocation	*MYB, MYBL1, NFIB, RAD51B*
Mitani et al., 2016 (32)	WGS, RT-PCR, FISH	Frozen Samples	Translocation	*MYB-NFIB*
			Rearrangement	*MYBL1-NFIB, MYBL1- YTHDF3*
Drier et al., 2016 (28)	WGS, RT-PCR, FISH	Frozen Samples, ACC primagrafts	Translocation	*MYB-NFIB*
			Rearrangement	*MYB-TGFBR3, MYB-RAD51B*
Rettig et al., 2016 (33)	FISH	FFPE	Translocation	*MYB-NFIB*
Tian et al., 2016 (61)	FISH	FFPE	Break	*MYB*
Rettig et al., 2016 (33)	WGS, RNA-seq	Frozen Samples	Deletion	*DUX4, DUX4L*
			Translocation	*MYB-NFIB*
			Fusion	*MAP3K5-NFIB, MYBL1-NFIB, NFIB-RPS6KA2, NFIB-MYO6, NFIB-RIMS1*
			Rearrangement	*NFIB*
Warner et al., 2018 (62)	WGS, RT-PCR	Primary culture	Translocation	*MYB-NFIB*

aCGH, Array comparative genomic hybridization; WGS, Whole genome sequencing; RNA-seq, RNA sequencing; FFPE, Formalin-Fixed Paraffin-Embedded; Primagrafts, primary patient-derived xenografts; **, Authors not discussing genes.

### Critical mutations

ACC has a low mutational load compared to other types of solid tumors. Despite this, some significant mutations have been reported, mainly in genes associated with cell signaling pathways in DNA damage response, growth factors, and chromatin remodeler genes (56, 63–65).

In relation to clinical-pathological features, missense mutations in *RAS* and *EGFR* are considered to be risk factors for DFS and overall survival (OS). In addition, *TP53* mutation was associated with expected overall survival in recurrent and metastatic ACC and is more frequent in the solid histological subtype (3). Mutations in *PI3KCA*, and *FGFR2* are also considered frequent, but there was no association with clinical-pathological findings (65, 66). However, both genes are particularly interesting due to their therapeutic potential (66).

Indeed, among the most well-described and important mutations in ACC are those that activate the NOTCH signaling pathway. Studies have shown that NOTCH knockdown is associated with the inhibition of growth, invasion (67) and cell proliferation (68) and that the deregulation of this pathway is associated with invasion and metastasis processes through the modulation of MMPs (metalloproteinases) and the induction of Epithelial-mesenchymal transition (EMT) (69). These findings are consistent with the fact that the *NOTCH1* mutation is more frequently observed in the solid histological subtype in metastatic tumors and is associated with a poor prognosis (64, 70, 71). The expression of *NOTCH* and its receptors may even partially explain the biphasic nature of low-grade and intermediate ACC. A study using single-cell RNA sequencing (scRNA-seq) demonstrated that luminal epithelial cells are more prone to the expression of *NOTCH* target genes as well as notch receptors, while DLL1, JAG1, and JAG2 ligands are more expressed in myoepithelial cells, suggesting paracrine signaling between these cell types (72).

Activation of the NOTCH pathway in ACC can also occur through mutations in other genes, such as *NOTCH2, NOTCH3, NOTCH4, SPEN, FBXW7* and *RBPJ* (40, 49, 50). Interestingly, ACC tumor samples with mutations in *NOTCH1* and *SPEN* had no alterations in *MYB* and *MYBL1*, suggesting that other genetic events than alterations in *MYB/MYBL1* may also play a central role in developing the ACC (32).

Strong evidence has suggested that epigenetic processes play an important role in the development of ACC. Mutations in chromatin remodeling genes are frequently reported (*SMARC1, SMARCA2, ARID1A, ARID1B, CREBBP, EP300*), and this class of genes has also been shown to be downregulated in ACC (*SMARCA4, SMARCB1, SMARCC1, SMARCC2, ARID1*, and *PBRM1*) (64, 71, 73, 74).

Chromatin remodeling is an essential process for gene expression, and changes in the chromatin landscape depend directly on the dynamics and density of nucleosomes, which are regulated by genes grouped into four categories: imitation switch (ISWI), chromodomain-helicase-DNA binding (CHD), inositol requiring 80 (INO80) and switching defective/sucrose nonfermenting (SWI/SNF). The activity of the SWI/SNF complex, in turn, depends on the activity of two mutually exclusive catalytic subunits, *SMARCA4* and *SMARCA2* (75).

The NOTCH signaling pathway can interact with chromatin remodelers through interactions with multiple proteins, mainly associated with the SWI/SNF complex. The catalytic subunit BRM is essential for ensuring the accessibility of chromatin to genes responsive to the NOTCH pathway (53). In this context, the BRM protein was shown to be overexpressed in ACC samples compared to normal salivary gland tissues. Interestingly, the genes encoding the other subunits of the SWI/SNF complex (INI1, BAF155, and BAF170) are downregulated, suggesting that the ACC presents an imbalance between the subunits of the SWI/SNF complex, which culminates in aberrant expression of several genes involved in cell cycle control, cell proliferation and adhesion (76). The simultaneous occurrence of mutations in chromatin remodelers and *NOTCH1* suggests that epigenetic mechanisms may promote progression by manipulating the NOTCH pathway through a possible biological synergy (65).

The patterns of gene expression were also described, and these findings culminated in the subdivision of ACC into subgroups according to the expression of *MYB/MYBL1*. In tumors that normally express these genes, their expression was positively correlated with genes associated with tumor proliferation (*EN1, PRAME, SOX4, SOX11, SMO, CDK6*, and *MYC*) (19, 54, 55). It is worth mentioning that *MYC* overexpression and advanced T stage were associated with lower DFS (29).

The frequency of activating mutations of the NOTCH pathway varies according to the methodology (65). It should be noted that studies that actually distinguish any mutation in *NOTCH* from activating mutations are limited (64, 71, 77). This distinction is important as a subpopulation of patients with activating mutations in this pathway could benefit from targeted therapies. In this sense, a complete analysis should encompass technologies that map other forms of NOTCH pathway activation and mutations in *NOTCH* 1/2/3. The combination of DNA-based NGS and NICD1 IHC is efficient in identifying patients with NOTCH pathway activation (64, 71, 77). The main limitation in defining a subgroup of patients who could benefit from targeted treatments remains the scarcity of data on staging classification by ICH, which makes it difficult to distinguish subgroups with activation of the pathway, and the lack of integration between clinical, pathological and genetic data (64, 71, 77).

On the other hand, there are subgroups of patients with ACC that do not express *MYB/MYBL1* express *KLF4, FOXO1, JUNB, FOSB, VGLL3* or *ERBB3, CTNNB1* and *SOX4*. The expression of *ERBB3, CTNNB1* and *SOX4* was associated with a survival of less than 10 years in patients with ACC (78).

## Targeted therapeutic strategies

**Figure 2:**
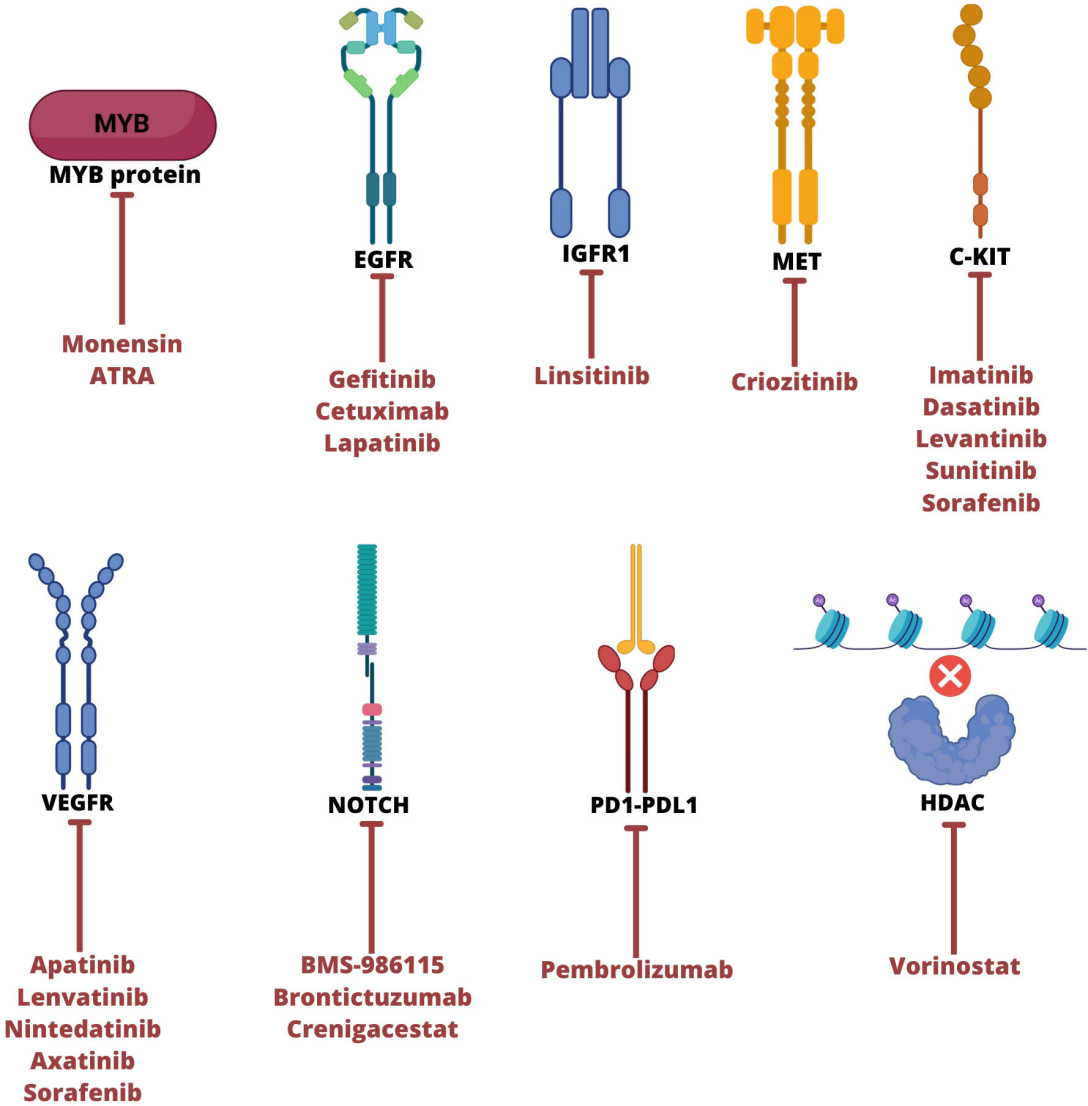
Main therapeutic targets to ACC tumor and their respective therapies. ATRA, all-trans retinoic acid; EGFR, epidermal growth factor receptor; IGFR1, insulin like growth factor 1 receptor; VEGFR, vascular endothelial growth factor; HDAC, Histone deacetylase. Created with BioRender.com.

Several studies have been developed to evaluate drug efficacy with targets in ACC. Clinical trials have been conducted to analyze patient progress by considering objective response rate (ORR/OR), stable disease (SD), progressive disease (PD), progression-free survival (PFS), or partial response (PR) according to RECIST criteria (66, 79–81). However, preclinical trials are limited due to the number of tumor samples available for experimental studies. *In vitro*, assays are also insufficient due to the few established cell lines, and some have proven cross-contamination (82). Finally, clinical trials rarely advance to phase 3, mainly due to insufficient objective responses (79–81). **Figure 2** and **Table 2** summarizes the target agents investigated in clinical trials for patients with ACC.

**Table 2:** Recurrent mutations in ACC.

Reference	Method	Gene
Ho et al., 2013 (3)	WGS	*PIK3CA, TP53, PTEN, SMARCA2, KDM6A, CREBBP, SMARCE1, ARID1A, ATRX, EP300, ARID4B, ARID5B, BRD1, FTSJD1, MLL, HIST1H2AL, HIST1H1E, UHRF1, TXNIP, ATM, BRCA1, DCLRE1A, NFIB, RYR3, RYR2, PTPRG, PTPRH, PTPRJ, PTPRK, FGF16, FGFR4, IGFBP2, ILR17RD, NOTCH1, FOXP2, DTX4, FBXW7, CNTN6, HSPG2, IDH1, NTNG1, SEMA3G, SEMA5A FAT3, FAT4*
Stephens et al., 2013 (65)	WES	*CDKN2A, PIK3CA, ATM, SUFU, TSC1, CYLD, SF3B1, NOTCH1, NOTCH2, ARID1A, CREBBP, EP300, KDM6A, MLL3, ARID1B, ARID1A, SMARCA2, CDH2, BRD2, KDM5A, SPEN, FGFR2*
Mitani et al., 2016 (32)	WGS	*CREBP, MUC12, TFAP4, MAP10*, *OTOP, NOTCH1*, *SPEN*
Ferrarotto et al., 2017 (64)	WES	*NOTCH1, NOTCH2*, *SPEN, NOTCH4, JAG1*, *FBXW7, RBPJ*
Andreasen et al., 2018 (16)	NGS	*NRAS*, *NOTCH1, BRAF, TP53, APC, PIK3CA, HRAS, PDGFRA, FGFR2*
Ho et al., 2019 (73)	WES, WGS and NGS	*NOTCH1*, *NOTCH2*, *NOTCH3*, *NOTCH4*, *SPEN*, *FBXW7*, *KDM6A*, *MLL3/KMT2C*, *ARID1B*, *ARID1A*, *BCOR*, *MLL2/KMT2D, CREBBP*, *ATM*, *LRP1B, TERT, MYB*, *MYBL1, SF3B1, XDH, LTF, TMEM2, MLH1, MSH6*
Adderley et al., 2021 (4)	NGS	*TP53, PI3KCA*
Ferrarotto et al., 2021 (83)	RNA-Seq	*NOTCH1, CREBBP, SPEN, EP300, RUNX1, SMARCB1, BCOR, ARID1B, KMT2C*

WGS, Whole Genome Sequencing; WES, Whole Exome Sequencing; NGS, Next Generation Sequencing.

### Inhibitors of MYB

Alterations in the *MYB* gene are considered a hallmark of ACC and have been a target in investigating new therapies for the disease. Recently, a study evaluated the antiproliferative activity of a small molecule ionophore inhibitor of MYB, monensin, in MYB-reporter cell lines. This study demonstrated a potential interference in ACC cell viability, downregulation of genes activated by *MYB-NFIB*, and a decrease in *MYB-NFIB* mRNA levels caused by this drug. These results suggest that monensin acts mainly on *MYB-NFIB* translocation, which could help develop a suppressor of cell proliferation (84).

Evidence of the frequent MYB-related abnormalities and the inhibitory potential of *MYB-NFIB* drugs *in vitro* encourage the development of clinical trials to evaluate this potential in ACC patients. Due all-trans retinoic acid (ATRA) appears capable of interrupting the self-regulatory enhancer loop that occurs with fusion genes involving MYB, decreasing the level of oncogenic fusion protein, a phase II clinical trial using this compound in patients with acute promyelocytic leukemia (APL) It was conducted (85). This is a provisional file, not the final typeset article had no objective response in ACC. On the other hand, a study in ACC population, 61% (11/18) of patients had SD, 28% (5/18) had PD as the best response, and no adverse effects grade ≥ 3 was observed. These results also suggest that the drugs mechanism of action does not appear to be related to MYB overexpression but provided a potential future direction for clinical use with other therapeutic schemes (86).

In general, chromosomal fusion results in overexpression of MYB, but inhibition of their coactivators decreases its activity. A previous study with linsitinib, crizotinib, and gefitinib that target IGFR1, MET, and EGFR decrease mRNA *MYB-NFIB* levels or even block protein synthesis, which promote cell differentiation and decrease of tumor growth *in vivo* and *in vitro*. This evidence implies a possible network between these genes. Recently, studies demonstrated that drugs that inhibit p300 and proteasome coactivators prevent MYB activation leading to the suppression of the proliferation of ACC lines (87, 88).

### Inhibitors of tyrosine kinase

The genetic alterations described in ACC impact relevant signaling pathways, including tyrosine kinase activity (48, 49, 63). One of the main targets studied in clinical trials is *C-KIT*. A relevant group of tyrosine kinase inhibitors (TKIs) evaluated against the protein-encoding this gene involves low molecular weight inhibitors. These types of TKI-specific target cancer cells by ligating transmembrane and intracellular proteins, affecting their enzymatic activity (89). Imatinib is a TKI targeting C-KIT, PDGFR, and ABL and has already demonstrated satisfactory activity in patients with chronic myeloid leukemia (CML) and gastrointestinal stromal tumors (GIST) (90–92).

After promising results of imatinib in GIST and CML, tumors that also overexpress C-KIT, studies have been conducted to investigate their clinical applicability in ACC. Reports using single imatinib therapy demonstrated limited responses, and none of the patients achieved OR, while the SD was not significant (75, 76). Additionally, dasatinib was well tolerated in ACC patients, but only 2.5% (1/20) had OR and 50% (20/40) had SD even with an experimental design that confirmed C-KIT overexpression (78). A phase II study using the combination of imatinib and cisplatin demonstrated that 11% (3/28) of patients evaluated had PR after induction chemotherapy (77). These findings, associated with the absence of mutations in exon 9 or 11 of *C-KIT*, suggest that only tyrosine kinase inhibitors may not be appropriate for patients with ACC (93–95).

EGFR is usually expressed in salivary glands, and their frequent activation in ACC tumors suggests that it may be a therapeutic target. Gefitinib is a drug capable of inhibiting EGFR in many tumors approved by FDA for treatment of locally advanced or metastatic non-small cell lung cancer (NSCLC). A phase II clinical trial with this drug unfortunately demonstrated no objective response, however, the drug was well tolerated, and there was prolonged SD and OS in some patients (96). Cetuximab, a monoclonal antibody that blocks the EGFR signaling approved for the treatment of colorectal cancer was used in ACC patients. In this phase II clinical trial demonstrated no objective response in ACC, but 87% (20/23) of patients had SD and 52% (12/23) had SD ≥ 6 months (97). Lastly, lapatinib, a double inhibitor of EGFR and erbB2, approved to be used on treatment of breast cancer HER 2+ was used in ACC patients and revealed no objective response, but 52% (15/19) of assessable patients had SD and 60% (9/15) had SD ≥ 6 months. These findings suggest that EGFR signaling is significantly active in ACC tumor and may be used as potential target for treatment of disease (98).

In addition to C-KIT and EGFR inhibitors, multitargeted medications, including other TKIs and inhibitor molecules of FGFR 1-4 signaling, VEGFR-1-3, RET, and PDGFR, have been studied. However, these studies are inconsistent about the best endpoint to evaluate the drug’s effectiveness in metastatic and/or recurrent ACC patients (99, 100). While a few authors defined SD and OR as the best criteria, others indicated that SD could not represent a real drug effect but instead the natural progression of tumor disease that has a long and indolent growth (66).

In this sense, lenvatinib and apatinib that were tested in phase II clinical trials demonstrated clinical efficacy in recurrent/metastatic ACC patients. Both studies established PFS as the primary endpoint, with durations of 9 and 19,8 months, respectively (66, 81). Conversely, other authors defined SD as the primary endpoint. Dovitinib, axitinib, and sunitinib demonstrated a better response in comparison with other TKIs in ACC tumors (SD = 65%, 75,8% and 78%, respectively), but no patient had OR (79, 90, 99).

The presence of FGFR activating mutations appears to be crucial for the satisfactory effect of dovitinib on other types of tumors (100). In ACC, a subpopulation that demonstrates activating mutations may benefit from this treatment, but this would need to be validated in larger cohorts as well as two-arm and randomized studies to observe the real effect of the drug (80).

The use of molecular markers could help to identify patients who may benefit from targeted drugs. For example, a longer median PFS was observed among MYB+/NFIB+ patients than among MYB+/NFIB-, MYB-/NFIB+ and MYB-/NFIB- patients. In addition, 3 cases of 4q12 amplification and, consequently, an increase in the copy number of the *PDGFRA/KDR/KIT* target genes were identified. In 2 of these 3 patients, axitinib produced tumor regression and SD for ≥ 6 months. Finally, the amplification of *NOTCH1* observed in 1 patient correlated with a confirmed partial response (cPR) with a PFS of nearly 1 year. Although these data are not statistically significant, they suggest that molecular investigation should also be considered in clinical trial designs (79).

### Cellular signaling pathway suppressors

Multi kinase inhibitors (MKI) and other drug types are currently used in clinical trials aiming to block signaling pathways. A multicenter phase II study evaluated the efficacy of everolimus, a mammalian target of rapamycin (mTOR) inhibitor that is important for proliferation signaling of the Akt pathway, in patients diagnosed with ACC in salivary glands. This study unfortunately demonstrated no complete or partial response (CR and PR) in patients with progressive unresectable ACC, but 79.4% (27/34) had SD, and tumor shrinkage within SD criteria was observed in 44% of these patients, showing a promising effect in advanced ACC patients (101).

Another drug used in clinical trials was nelfinavir, a human immunodeficiency virus (HIV) protease inhibitor that is generally used in acquired immunodeficiency syndrome (AIDS) patients. In cancer, this drug suppresses the PI3K/Akt/mTOR signaling pathway and a previous study evaluated this drug in ACC patients including salivary glands and the best response was SD in 46.7% (7/15) of patients, and no patients had PR or CR (102).

### Inhibition of NOTCH pathway

The NOTCH signaling is essential for ACC progression and support the stem cell characteristic maintenance and angiogenesis. A phase I clinical trial testing the BMS-986115 (pan-NOTCH inhibitor) in 36 tumor solid patients including ACC revealed two ACC cases with SD ≥ 6 months, and the author suggest that this drug can interfere actively in ACC progression (103). In addition, the use of brontictuzumab, a monoclonal antibody that blocks NOTCH1 pathway, demonstrate an effective action in ACC patients. A phase I study showed the efficacy of this drug in many cancer patients including ACC with PRs and three prolonged SD occurred in ACC subjects with evidence of *NOTCH1* pathway activation (104).

Further, a multicenter phase I clinical trial evaluated the safety and toxicity of crenigacestat (LY3039478) monotherapy, a small molecule inhibitor of NOTCH signaling. In this study, 68% of patients (15/22) had SD and four had SD ≥ 6 months. AE were mild to moderate and some patients had AE grade ≥ 3, including diarrhea 3 (14%), fatigue 1 (5%) and ALT increase 1 (5%) (105).

### Antiangiogenic therapies

Angiogenesis develops a crucial role in ACC progression and many genes are required for this process. A multicenter phase II study demonstrated no objective response using nintedatinib, a triple TKi including VEGFR, FGFR and PDGFR. However, this drug was had tolerable toxicity and a high rate of disease control and stabilization in patients with recurrent or metastatic salivary gland cancer with majority of ACC cases. In addition, levantinib demonstrated interesting results in a phase II study: 5 of 33 patients had partial response, 24 had SD and only one had PD. Therefore, 66% (21/32) had tumor regression and 25% (8/32) had 20% or greater of tumor shrinkage, however, AE grade ≥ 3 were reported including hypertension and oral pain (106).

A clinical trial phase II evaluated the safety and efficacy of axitinib in salivary gland tumors including ACC. This drug consists of inhibitor of VEGFR 1, 2 and 3 and it is approved for treatment of advanced renal cell carcinoma. The study showed a tumor shrinkage and SD in ACC patients (107). A European phase II study evaluated sorafenib, a multitarget inhibitor of VEGFR, cKIT, p38a, PDGFRb in ACC population. Majority of patients (68%) had SD and unfortunately 21% had PD. Moreover, the toxicity was frequent (108). Other phase II study using sorafenib in head and neck cancer including ACC patients demonstrate 2 of 19 patients had partial response and 9/19 had stable disease. These findings indicate the limited effect of antiangiogenic therapies (109).

### Immune targets

Understanding the tumor immune microenvironment is essential to provides biomarkers to develop novel target therapies. Lymphocytic infiltration supports the immune response against tumor cells and have been associated to favorable prognosis and the expression of molecules associated with immune infiltration are variable and ACC usually presents low immunogenicity (12). Recently, a pioneer phase II clinical trial evaluated the safety and OR of pembrolizumab plus vorinostat in head and neck squamous cells and salivary gland cancer patients (110). Pembrolizumab consists of a humanized mouse- derived PD-1 (programmed cell death protein 1) antibody that interrupt programmed death-ligand 1 (PD-L1) ligation promoting tumor cells apoptosis. FDA approved pembrolizumab to for use in patients with melanoma, head and neck squamous cell carcinoma, classical Hodgkin lymphoma and others solid tumors. On the other hand, vorinostat corresponds to a histone deacetylase (HDAC), that improves cell cycle arrest, apoptosis and angiogenesis inhibition approved to cutaneous T-cell lymphoma treatment (111). In this study with both drugs, 16% (4/25) of patients had PR and 40% (10/25) patients had SD ≥ 6 months. They observed stronger toxicity than studies with only pembrolizumab (110).

Finally, a phase II clinical trial of pembrolizumab, an immunotherapy drug potentially blocking PD-1 was conducted. In this study, the effects of medication accompanied by radiation (Arm A) and single medication therapy (Arm B) were evaluated. The SD was the best response observed among 50% (5/10) of patients in arm A and 70% of patients (7/10) in arm B without statistical significance, and no participants presented OR (112). More details of the current clinical trials involving ACC are summarized in **Table 3**.

**Table 3:** Clinical trials for the ACC.

Drug	Trial identifier	Target	Phase	SD %	PFS	Publication
Sunitinib	NCT00886132	A multi-targeted RTKi, including VEGFR 1–3, c-kit, PDGFR-α/β, RET and FLT3	Phase II	62%	6 months	Chau et al., 2012 (99)
Everolimus	NCT01152840	PI3k-mTOR pathway inhibitor	Phase II	79,4%	11.2 months	Kim et al., 2014 (101)
Gefitinib	NCT00509002	EGFR	Phase II		4.3 months	Jakob et al., 2015 (96)
Dasatinib	NCT00859937	Inhibitor of five oncogenic PTKs/kinase families including cKIT	Phase II	50%	19.2 weeks	Wong et al., 2016 (95)
Nelfinavir	NCT01065844	Suppressing Akt signaling	Phase II	46,66%	5.5 months	Hoover et al., 2015 (102)
Axitinib (AG-013736)	NCT01558661	RTKi of VEFGRs 1–3, KIT and PDGFRs A/B	Phase II	75,8%	5.7 months	Ho et al., 2016 (79)
Sorafenib	NCT01703455	VEGFR 1-3, BRAF, PDGFR, RET, and C-KIT	Phase II	59%	8.9 months	Locati et al, 2016 (109)
Dovitinib	NCT00581360	A multikinase inhibitor that suppresses FGFR 1-3 signaling	Phase II	65%	8.2 months	Dillon et al., 2017 (80)
Brontictuzumab	NCT01778439	NOCTH1	Phase I	28%	65 days	Ferrarotto et al., 2018 (104)
Pembrolizumab	NCT03087019	Block PD-L1	Phase II		4.5 months (Arm A) 6.6 months (Arm B)	Mahmood et al., 2021 (112)
	NCT02538510		Phase II	16%	6.9 months	Rodriguez et al., 2020 (110)
Lenvatinib	NCT02860936	TKi, targeting VEGFR-1-3, FGFR-1-4, RET, c-KIT, and PDGFR	Phase II			Locati et al., 2020 (81)
All-trans retinoic acid (ATRA)	NCT03999684	A inhibitor of MYB		61%	3.2 months	Hanna et al., 2021 (86)
Apatinib	NCT02775370	VEGFR blocker	Phase II	Not provide	19.7 months	Zhu et al., 2021 (66)

PFS, Progression Free Survival; SD, Stable Disease.

## Conclusion

Despite being a rare disease, ACC is a very aggressive tumor, and patients frequently exhibit perineural invasion and distant metastases due to molecular alterations. In this sense, t(6;9) translocation, resulting in *MYB-NFIB* fusion, is the most frequently reported alteration in ACC. Additionally, translocations involving *MYBL1* and other enhancer genes were observed. This alteration results in MYB protein overexpression, which has several activation mechanisms, highlighting the complexity of this tumor.

Chromosomal gains and losses also affect important genes that are responsible for several signaling pathways and cell proliferation, such as NOTCH and C-KIT. Interestingly, the majority of mutations in ACC do not occur simultaneously at t(6;9), suggesting that ACC has a multistep initiation and progression process. Even epigenetic changes are involved in this tumor biology, such as key mutations in chromatin remodeling (*MERC1, SMARCA2, ARID1A, ARID1B, SMARCA4*) and an imbalance in miRNAs (miR-155, miR-320a, miR-99a, miR-205) that regulate gene expression.

The described genetic alterations have been constantly investigated as possible targets for new therapies. However, no targeted therapy has demonstrated consistent effectiveness, and ACC treatment currently still includes surgery and postoperative radiotherapy, which do not have an effect in metastatic patients. Natural blockers of MYB overexpression have demonstrated promising results in leukemia; however, in ACC patients, preclinical investigation is difficult due to the lack of cell lines and *in vivo* models.

To try to reverse this situation, studies with significant cohorts are needed, as well as the development of *in vitro* models. In this sense, 3D cultures would be interesting to analyze the impact of the tumor microenvironment on the ACC. The identification of molecular subtypes with specific alterations would also be interesting in clinical practice for the selection of patients who may benefit from certain therapeutic strategies. But, for that, it is necessary to standardize the methodologies used for the detection of important molecular alterations.

Finally, the authors generally report challenges in evaluating drugs with antitumor activity, since due to the rarity of the disease, it is difficult to establish a homogeneous population regarding histological type, previous therapy and metastatic disease. Apparently, RECIST criteria may not be useful to assess drug efficacy because of slow tumor growth. Therefore, it is recommended that efforts be focused on multicenter studies to improve patient selection (matching according to histological subtype, presence/absence of driver mutations and other relevant clinicopathological criteria) and the design of clinical trials.

## Author contributions

Study design: FS and DC. Drafting of the manuscript: FS, JJ, and DC. Tables and Figures: FS and JJ. Manuscript review: AR, JP, and VF. All authors contributed to the article and approved the submitted version.
